# Long post-COVID-vaccination syndrome manifesting as temperature-sensitive myalgia and hyper-CKemia

**DOI:** 10.1016/j.clinsp.2023.100175

**Published:** 2023-03-06

**Authors:** Josef Finsterer, Carla A. Scorza, Fulvio A. Scorza

**Affiliations:** a Neurology & Neurophysiology Center, Vienna, Austria; b Disciplina de Neurociência, Universidade Federal de São Paulo, Escola Paulista de Medicina (UNIFESP/EPM), São Paulo, SP, Brasil

**Keywords:** BPV, Biontec Pfizer vaccine, CK, creatine-kinase, CNS, central nervous system, COVID, coronavirus disease, ECG, electrocardiogram, EMG, eElectromyography, MIS-V, multisystem inflammatory syndrome after vaccination, NMDs, neuromuscular disorders, PNS, peripheral nervous system, SARS-CoV-2, severe, acute respiratory syndrome-coronavirus-2

There is growing evidence that SARS-CoV-2 vaccines are not safe for everyone.^1^ Since the introduction of the SARS-CoV-2 vaccination in December 2020 numerous cases of side effects after the first, second, third, or fourth injection have been reported in the literature or on various online platforms.^2^ Among the reported side effects, those manifesting in the Central or Peripheral Nervous System (CNS, PNS) outweigh those affecting the heart, endocrine organs, kidneys, or skin.^1^ One of the PNS tissues occasionally affected is the skeletal muscle.^3^ Side effects manifesting in the skeletal muscles include myositis,^4^ dermatomyositis, immune-mediated necrotizing myopathy,^3^ rhabdomyolysis, and polymyalgia rheumatic, A patient with temperature-dependent myalgias after the third jab with the Biontech Pfizer Vaccine (BPV) has not been reported.

The patient is a 34-year-old female who developed dizziness 30 minutes after the third BPV injection followed by a fever of up to 39°C. Six days later, she developed generalized myalgias and precordial chest pain, and suffered from previously unknown fatigue and exercise intolerance. She was no longer able to do her usual exercises at the gym she attended regularly prior to the vaccination. Fever went back spontaneously but always, slightly increased body temperature and dizzy spells have been noticed since then. The myalgias were highly temperature sensitive, such that cold increased myalgias while the heat produced transient relief of myalgias. Exposure to cold for >20 minutes even led to immobility to move. The patient tolerated the first BPV injection without any major complications, but after the second BPV dose she had a fever, tiredness, exhaustion, and polymyalgias, some of which resolved spontaneously after a few days. Although she was able to return to work, fatigue, and exhaustion persisted for weeks.

Her history was positive for the cervical syndrome, dysmenorrhoea, depression, anxiety disorder with panic attacks, smoking (10 cigarettes per day), and multiple allergies. The only medication she took regularly was an anti-contraceptive shot every three months. Family history was negative for neuromuscular disorders (NMDs).

The clarification of the myalgia revealed general muscle soreness, increased creatine-kinase (CK) up to 1063 U/L (n < 170 U/L), elevated myoglobin up to 64.4 ng/mL (n < 47 ng/mL), but normal C-reactive protein, blood sedimentation rate, and thyroid function tests. The patient refused to undergo needle electromyography (EMG) and muscle biopsy due to needle phobia. Muscle MRI with contrast medium was inconclusive. A workup of anginal chest pain revealed a normal clinical examination, normal blood pressure, normal ECG, normal echocardiography, normal CK-MB, normal troponin-C, and normal proBNP, therefore myocarditis was largely ruled out. At the last follow-up 10 weeks after the third BPV, myalgia was still present, but CK had decreased to 581 U/L. Analgesics provided only temporary relief, but the patient refused to undergo attempted glucocorticoids, intravenous immunoglobulins, or plasmapheresis.

This case shows that SARS-CoV-2 vaccinations can be harmful even for patients without serious comorbidities, that SARS-CoV-2 vaccinations can cause muscle damage with temperature-sensitive myalgias, and that these symptoms can last for weeks.

Myalgia is a common complication of SARS-CoV-2 vaccinations, for example occurring in 50% of vaccinees after the first BPV dose and in 75% of the vaccinees after the second dose.^5^ Myalgia occurs less frequently after the third dose compared to the first and second doses of BNV.^6^

The cause of myalgia remains elusive but given previous reports of immunological side effects.^7^ it is conceivable that myalgia may result from a cross-reaction of the immune system against the vaccine and skeletal muscle. Other pathophysiological mechanisms could be the production of autoantibodies or the immunogenic role of certain vaccine adjuvants.^7^ A strong argument for an immunogenic cause of myalgia is the fact that it usually resolves after steroid administration.^8^

Whether the patient had post-vaccination multisystem inflammatory syndrome after Vaccination (MIS-V) in an adult, as recently reported,^9^ remains controversial. Hyper-CKemia has been previously reported as a manifestation of MIS-V, but these patients also have a multisystem disease including fever, diarrhea, abdominal pain, rash, or edema.

Whether SARS-CoV-2 vaccine-induced myalgia is indicative of subclinical myopathy cannot be ruled out but those with a hereditary myopathy typically do not report adverse reactions to COVID vaccines more frequently than those without a hereditary myopathy. However, one patient with an RYR1 mutation developed rhabdomyolysis after the second dose of the Moderna vaccine.^10^

In summary, SARS-CoV-2 vaccines are not safe for everyone and can lead to long-term impairment, disability, and social decline. Manufacturers and health authorities are urged to provide SARS-CoV-2 vaccines that are safe for everyone.

## Declarations

Ethics approval: Was in accordance with ethical guidelines. The study was approved by the institutional review board.

Consent to participate: Was obtained from the patient.

Consent for publication: Was obtained from the patient.

Availability of data: All data are available from the corresponding author.

## Code availability

Not applicable.

## Authors' contributions

Josef Finsterer: Design, literature search, discussion, first draft, critical comments, final approval. Carla A. Scorza and Fulvio A. Scorza: Literature search, discussion, critical comments, final approval.

## Declaration of Competing Interest

The authors declare no conflicts of interest.
